# Supplementary material for: Earlier Morning Arrival to Pollen-Rewarding Flowers May Enable Feral Bumble Bees to Successfully Compete with Local Bee Species and Expand Their Distribution Range in a Mediterranean Habitat
Source: Insects. 2022 Sep 7;13(9):816. doi: 10.3390/insects13090816 (PMC9503872; doi:10.3390/insects13090816)
Supplement: Supplementary file 1 [file insects-13-00816-s001.zip › insects-1834176-supplementary.pdf]

Supplementary Materials

Earlier morning arrival to pollen rewarding flowers may enable feral bumble bees to successfully compete with local bee species and expand their distribution range in a Mediterranean habitat

Noam Bar Shai[1,2*], Uzi Motro[1,3,4], Avishai Shmida[1,4] and Guy Bloch[1,4*]

1. Department of Ecology, Evolution and Behavior, The A. Silberman Institute of Life Sciences, The Hebrew University of Jerusalem, Jerusalem, Israel.
2. Jerusalem Botanical Garden, The Hebrew University of Jerusalem, Jerusalem, Israel
3. Department of Statistics, The Hebrew University of Jerusalem, Jerusalem, Israel
4. The Federmann Center for the Study of Rationality, The Hebrew University of Jerusalem, Jerusalem, Israel

Survey of *B. terrestris* range on the western and southern margins of the Judean hills.

On July 2012 and June 2014, we made excursions to assess the current distribution range of *B. terrestris* in the Judean hills. We focused on the western and southern margins since *B terrestris* spreads southwards (Dafni & Shmida 1996, see Introduction). Therefore, the northern margins are considered to already lie within its range, and eastwards the climate changes rapidly into the hot and dry Judean Desert, where *B. terrestris* has never been reported. Towards the west, the elevation decreases gradually and the climate becomes warmer, and towards the south the climate becomes gradually warmer and drier. We focused our surveys on June and July when bumble bees are abundant (Fig. 2).

On July 2012 we drove southwards along the "mountain range road" (route no. 60) and looked for bumble bees on public gardens. We found bumble bees at Alon-Shvut, Kiryat-Arba and Beit-Haggay, but none at Otni'el and southwards (Supplementary Table S1, Supplementary Figure S1). On June 2014 we performed several excursions towards the Shefela plain which lies west of the Judean Hills. We focused on *Vitex agnus-castus* plants that grow along streams (mostly dry), since it has been found to be the main forage plant in this season, and it is abundant along such streams. We assumed, therefore, that where bumble-bees are present, they will be found on *V. agnus-castus*. In most streams, we found bumble-bees at the upper, eastern parts of these streams, but they were not spotted anymore when we further continued westward (Supplementary Table S2, Supplementary Figure S1). The westernmost points were at approximate altitudes of ~200m above sea level in Nachshon & Soreq streams, 270 m in Ha'Ella stream which lies south of them, and ~330m at Guvrin stream which is the southernmost. An additional excursion was made to on the same month to Amatzia area at the southern Shefela plain. No *V. agnus-castus* plant were found, but bumble-bees were absent from *Verbascum sinuatum* which was very abundant there and appeared to be among the major forage plants (pollen) of *B. terrestris* at Kennedy and Soreq transects. The southernmost report of *B. terrestris* was from a site east of Beth Guvrin, at 31.600092, 34.938303, also on June 2014. The visited plant was not identified by the reporter (Figure S1, point L, Ron Bosak - personal communication).

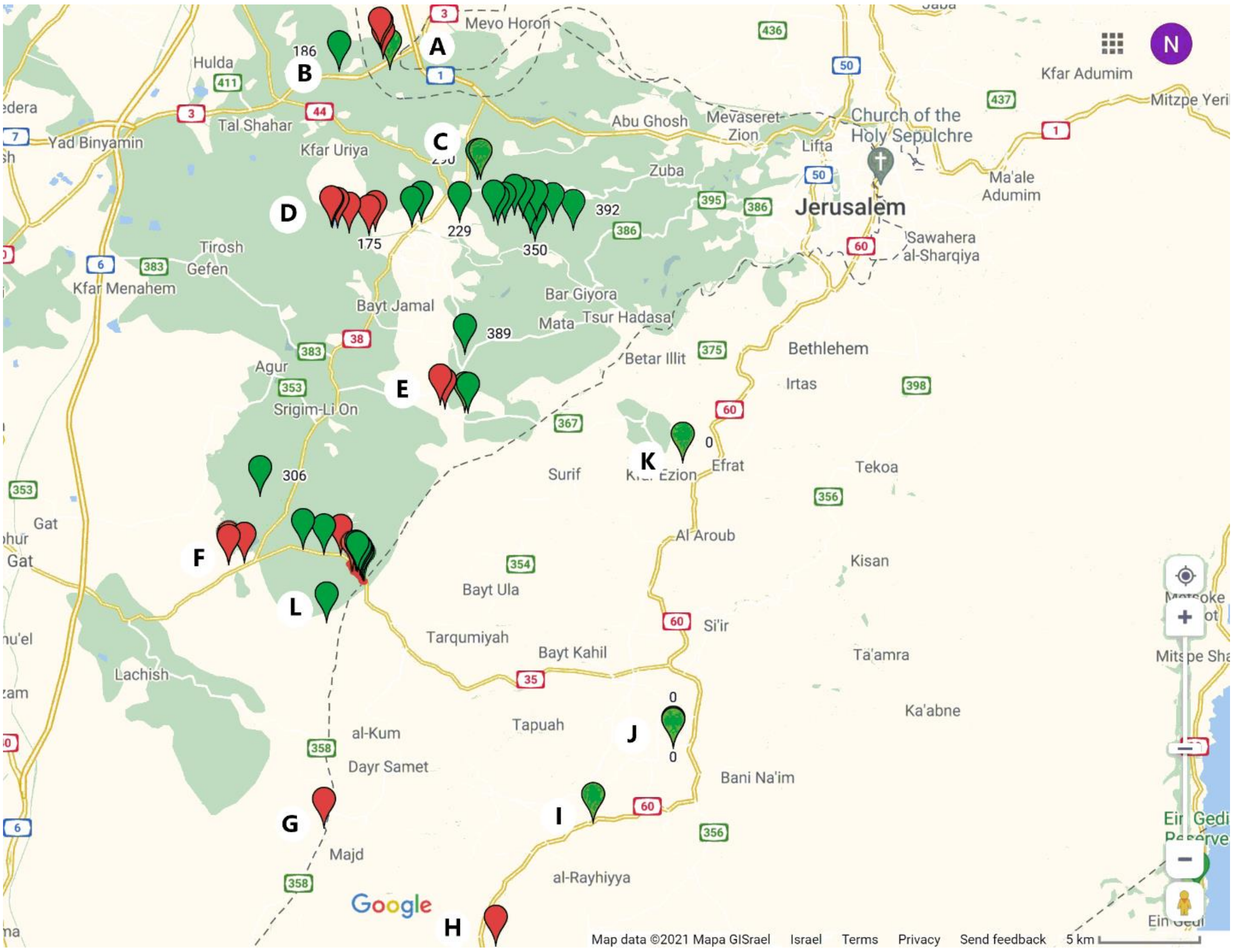

Figure S1. Presence of *B. terrestris* at western and southern margins of Judean Hills on July 2012 and June 2014 surveys, green and red symbols are locations in which bumble bees were present or absent, respectively; A - Me'ir/ Nachshon stream; B - Kibbutz Nachshon Junction; C - Kesalon stream; D – Soreq stream; E – Ha'Ella Stream; F - Guvrin Stream; G – Amatzia area; H – Otni'el; I – Beth-Haggai; J – Kiryat-Arba; K – Alon-Shvut; L – east of Beth-Guvrin;.

Table S1. Presence of *B. terrestris* south of Jerusalem along route no. 60 on July 2012. Sites are arranged from north to south.

	site	Altitude	Latitude	Plants	Presence
**1**	Alon-Shvut	950	31.66° N	Public gardening	Present
**2**	Kiryat-Arba	940	31.53° N	Lavandula sp.	Present
**3**	Beth-Haggai	850	31.49° N	Lavandula sp.	Present
**4**	Otni'el	700	31.44° N	Public gardening	Absent
**5**	Shim'ah	620	31.39° N	Various plants	Absent
**6**	Carmel	750	31.43° N	Public gardening	Absent

Table S2. Presence of B. terrestris at western margins of Judean Hills on June 2014. Sites are arranged from north to south. Asterix denotes sites where bumblebee were present but there was no continuous range of *Vitex agnus-castus* that could be followed. On other sites, the altitude & longitude denotes the points where no bumble-bees were observed west of them.

	stream	Altitude	Longitude	Plant	comments
**1**	Nachshon stream	190	34.98° E	V. agnus-castus	Mostly dry
**2**	Kibbutz Nachshon junction *	160	34.95° E	Cultivated V. agnus-castus	Not a stream
**3**	Kesalon stream*	270	35.02° E	V. agnus-castus	Dry
**4**	Soreq stream	200	34.99° E	V. agnus-castus	High flow of purified sewage
**5**	Ha'Ella stream	270	34.95° E	V. agnus-castus	Dry
**6**	Guvrin stream	330	34.95° E	V. agnus-castus	Partly dry

Table S3. Blooming timeline, in increments of half-months, of plants visited by *B. terrestris* in Soreq site during 2012, 2013 & 2014 seasons. Pink and yellow backgrounds denote plants in which the major floral reward is nectar or pollen, respectively.

	Apr2	May1	May2	Jun1	Jun2	Jul1	Jul2	Aug1	Aug2	Sep1
Antirrhinum majus	+	+	+	+						
Satureja thymbra	+	+	+	+						
Salvia fruticosa	+	+	+	+	+	+				
Cistus creticus	+	+	+	+	+	+				
Alcea setosa		+	+							
Hypericum languinosum		+	+	+						
Rosa phoenicia		+	+	+						
Teucrium creticum		+	+	+	+	+				
Capparis zoharyi		+	+	+	+	+	+			
Melissa officinalis			+	+	+					
Verbascum sinuatum			+	+	+	+	+	+	+	+
Echinops adenocaulus				+	+	+	+	+		
Vitex agnus-castus				+	+	+	+	+	+	+
Carthamus tenuis							+	+	+	
